# High-efficiency nonhomologous insertion of a foreign gene into the herpes simplex virus genome

**DOI:** 10.1099/jgv.0.001451

**Published:** 2020-06-30

**Authors:** Yue Gong, Yanwei Bi, Zhihua Li, Yuzhong Li, Yueting Yao, Qiong Long, Tao Pu, Chen Chen, Tongyun Liu, Shaozhong Dong, Wei Cun

**Affiliations:** ^1^​ Institute of Medical Biology, Chinese Academy of Medical Sciences & Peking Union Medical College, Kunming 650118, Yunnan, PR China; ^2^​ Yunnan Key Laboratory of Vaccine Research and Development of Severe Infectious Disease, Kunming, 650118, Yunnan, PR China; ^3^​ The First Affiliated Hospital of Kunming Medical University, Kunming 650118, Yunnan, PR China

**Keywords:** Herpes simplex virus, CRISPR/Cas9, gene knock-in, nonhomologous end joining

## Abstract

Efficient, accurate and convenient foreign-gene insertion strategies are crucial for the high-throughput and rapid construction of large DNA viral vectors, but relatively inefficient and labour-intensive methods have limited the application of recombinant viruses. In this study, we applied the nonhomologous insertion (NHI) strategy, which is based on the nonhomologous end joining (NHEJ) repair pathway. Compared to the currently used homologous recombination (HR) strategy, we obtained a higher efficiency of foreign-gene insertion into the herpes simplex virus (HSV) genome that reached 45 % after optimization. By using NHI, we rapidly constructed recombinant reporter viruses using a small amount of clinical viruses, and the recombinant virus was stable for at least ten consecutive passages. The fidelity of NHI ranged from 70–100% and was related to the sequence background of the insertion site according to the sequencing results. Finally, we depict the dynamic process by which the foreign-gene donor plasmid and viral genome are rapidly cleaved by Cas9, as revealed by quantitative pulse analysis. Furthermore, the NHI strategy exerted selection pressure on the wild-type and reverse-integrated viral genomes to efficiently integrate the foreign gene in a predetermined direction. Our results indicate that the use of a rationally designed NHI strategy can allow rapid and efficient foreign gene knock-in into the HSV genome and provide useful guidance for gene insertion into large DNA viral genomes using NHI.

## Introduction

Viral vectors are a useful tool for the delivery of foreign genes into cells [1], and herpesvirus can be applied in fields such as gene therapy and the development of oncolytic vectors and replication-deficient vaccines due to its large genome capacity (encoding a variety of nonessential genes) and because it causes minor pathological damage [2]. Compared to relatively small viral genomes (such as those of adeno-associated virus and lentivirus) in which the insertion of a foreign gene can be carried out by the restriction-ligation method, the insertion of a foreign gene into a large viral genome that exceeds 100 kb demands host cellular homologous recombination (HR) or the construction of a bacterial artificial chromosome (BAC) [3–5]. These inefficient, time-consuming and labour-intensive strategies have hampered the development and promotion of large DNA viral vectors.

The clustered regularly interspaced short palindromic repeats/CRISPR-associated protein 9 (CRISPR/Cas9) system, in which the Cas9 protein can target and cleave the genome in a sequence-specific manner facilitated by guide RNA (gRNA), resulting in a double-strand break (DSB), was established in 2013 [6–9]. Furthermore, DSBs induce two primary DNA repair mechanisms, nonhomologous end joining (NHEJ) and HR, by which genome-specific gene knockout, site-directed mutagenesis and the insertion of foreign genes are carried out. Because of its advantages (its simplicity, efficiency and multisite targeting), the CRISPR/Cas9 system has been widely applied to modify the genomes of different species [10, 11]. The CRISPR/Cas9 system not only simplifies the procedure of genome editing but also significantly improves the efficiency of genome editing.

In 2014, we first used CRISPR/Cas9 to genetically edit the episomal DNA viral genome in mammalian cells [12]; use of the CRISPR/Cas9 system increased the efficiency of gene replacement in large viral genomes by at least 10 000-fold compared to other strategies [13]. Due to highly efficient viral-genome replication, we observed the inhibitory effect of CRISPR/Cas9 on replication of the genome, which contained a target sequence specifically cleaved by the CRISPR/Cas9 system. By using such sequence-specific selection pressure for the screening of recombinant viruses, the efficiency by which point mutations were made in the viral gene was increased to more than 50 % [14]. However, for the insertion of large foreign genes into large viral genomes, the HR-mediated gene insertion efficiency is significantly lower than the NHEJ-mediated gene knockout efficiency [15], and the larger the foreign gene is, the more difficult the screening for recombinant virus is. Therefore, it is necessary to further improve the efficiency of foreign-gene insertion into viral genomes.

NHEJ is a more efficient mechanism of cellular DNA repair than HR [16]. When we first used NHEJ for gene knockout, NHEJ was considered to be an error-prone DNA repair method. However, in recent years, there has been increasing evidence indicating that the indel produced by NHEJ might be a repair outcome of cutting pressure applied by CRISPR/Cas9. In other words, the tendency of NHEJ to carry out imprecise DNA repair may be amplified by iterative cycles of the cut-and-repair process. Therefore, rational design of the foreign-gene junction sequence can avoid a CRISPR/Cas9-mediated cut after NHEJ-mediated insertion of the foreign gene into the viral genome, which not only improves the efficiency of directional insertion but also reflects the true fidelity of NHEJ in DNA repair. An NHI strategy with the same design that can efficiently insert foreign fragments into nondividing mammalian cells has been developed [17]. However, unlike the relatively slow replication cycle of the eukaryotic genome, the viral genome can undergo rapid multicopy replication in the cell. In addition, there are only approximately two target sites in the eukaryotic genome in a single diploid cell, but target sites in the viral genome can be exponentially produced during viral infection. Therefore, the cutting and selection pressure applied by the CRISPR/Cas9 system to the viral genome, as well as the repair rate and fidelity of NHEJ-mediated insertion, have yet to be evaluated.

This study utilized CRISPR/Cas9 system-mediated cutting and selection pressure in conjunction with an NHEJ-mediated insertion strategy and optimized experimental conditions to further increase the efficiency of foreign-gene insertion into the herpesvirus genome in host cells. Furthermore, we applied quantitative pulse analysis to describe the dynamic cut-and-insertion process in the NHEJ-mediated insertion method. Overall, we aimed to establish a more rapid and simpler method of directional foreign-gene insertion into a large DNA viral genome.

## Methods

### Cell cultures and virus

The African green monkey kidney Vero cell line (ATCC, Manassas, VA, USA) was cultured in minimum Eagle’s medium (MEM; Thermofisher Scientific) supplemented with 10 % FBS (HyClone). Human embryonic kidney 293T (HEK-293T) cells were cultured in Dulbecco’s modified Eagle’s medium (DMEM; Thermofisher Scientific) supplemented with 10 % FBS. All media were supplemented with 10 % 100 U ml^−1^ penicillin and 100 mg ml^−1^ streptomycin. All cells were maintained at 37 °C with 5 % CO_2_. After infection for 2 h, the cells were cultured in MEM or DMEM supplemented with 2 % FBS. The laboratory passage HSV1-8F strain (12) and clinically isolated HSV1-ZW6A strain (GenBank: KX424525.1) were amplified and titred on Vero cells. The viral genome was extracted from clinical isolates as previously described [18], and next-generation sequencing was performed with PacBio by NextOmics (Wuhan, Hubei, PR China). For the serial passage of recombinant virus, 6×10^6^ Vero cells were infected with 500 µl of harvested virus for 2 h at 37 °C, the supernatant was then discarded, and the cells were washed with PBS once and incubated with 10 ml of DMEM containing 10 % FBS. After 24 h, the progeny virus was harvested and stored at −80 °C.

### Plasmid construction and minicircle donor production

Cas9 target sites in the HSV1 genome were analysed using the online tool CRISPOR to exclude potential off-target sites [19]. Cas9: gRNA bicistronic expression plasmids were constructed as previously described [12]. The oligos used for construction Cas9: gRNA bicistronic expression for site 2,3,4,5 were annealed and ligated into linear plasmid, and the Cas9: gRNA bicistronic expression plasmid for site 1 was described before [12].

To construct HR donors, the pUC19 vector was cut with EcoRI and BamHI (New England BioLabs) and used as the backbone. Then, 800–1000 bp homologous sequences flanking the Cas9 cut site of the HSV genome were amplified from the HSV1-8F genome, and the EGFP cassette was amplified from the pEGFP-N2 plasmid. Finally, the two homologous sequences and EGFP cassette were ligated simultaneously into the pUC19 vector using the ClonExpress MultiS One Step Cloning Kit (Vazyme).

For the NHI 2 c donor, the EGFP cassette gene was amplified, and a 23 bp Cas9 cut site was introduced simultaneously with the oligo pair using PCR and then ligated to the backbone plasmid using a pMD19-T Vector Cloning Kit (TaKaRa), and the upper oligo pairs used for site 1 was N151 (5′- CCGACGTACGGCGTTGCGCCCTCCGTTACATAACTTACGGTAAATGG −3′), the lower oligo was N152 (5′- GAGGGCGCAACGCCGTACGTCGGTAAGATACATTGATGAGTTTGGACA −3′); for site 2 was N390 (5′- CCACGTCGAAGGCTTTTGCATTGCGTTACATAACTTACG −3′) and G220 (5′- CAATGCAAAAGCCTTCGACGTGGTAAGATACATTGATGAGTTTGGACA −3′); for site 3 was N367 (5′- CGTGACAAAACGGACCCCCTTGGGCGGCCGCCGTTACATAACTTACGGTAAATGG −3′) and N368 (5′- CCAAGGGGGTCCGTTTTGTCACGGCTAGCTAAGATACATTGATGAGTTTGGACA −3′); for site 4 was N363 (5′- CCGCGGGGGATCGATAATTCGCCGCGGCCGCCGTTACATAACTTACGGTAAATGG −3′) and N364 (5′- GGCGAATTATCGATCCCCCGCGGGCTAGCTAAGATACATTGATGAGTTTGGACA −3′); for site 5 was N389 (5′- CGCGACCTAACAAAACCCGGGGGCGTTACATAACTTACG −3′) and G221 (5′- CCCCCGGGTTTTGTTAGGTCGCGTAAGATACATTGATGAGTTTGGACA −3′); for the NHI 1 c donor, the upper oligo was the same as that used for the 2 c donor, and the lower oligo was N155 (5′-TAAGATACATTGATGAGTTTGGA-3′). For site 6, the upper oligo was N489 (5′-CCCCCATCTCCCGGGCAAACGTGATGGTGAGCAAGGGCG-3′), the EGFP cassette without the Cas9 cut site was amplified and ligated to construct the NHI 0 c donor with oligos N156 (5′-CGTTACATAACTTACGGTAAATGG-3′) and N155.

To construct the minicircle donor plasmid, the backbone was amplified from the pMC.CMV-MCS-EF1α-GFP-SV40polyA Parental Minicircle Cloning Vector (System Biosciences) using oligos N157 (5′-CGGAATTCCATCGACCCATGGGGGC-3′) and N158 (5′-GCGGATCCAGTATCAATTCACTAGTCGCGC-3′). Then, the EGFP cassette with the 23 bp cut site was amplified from the corresponding NHI 1 c donor plasmid and ligated into the backbone using the ClonExpress II One Step Cloning Kit (Vazyme). For site 1, the oligo pairs used were N159 (5′-GATCCAGATCTGATATCTCTAGAGTCGACG-3′) and N160 (5′-AATTCGTCGACTCTAGAGATATCAGATCTG-3′), and for sites 2 and 5, the oligo pairs N163 (5′-TGAATTGATACTGGATCCGCCCCGGGGATCCTCTAGAG-3′) and N164 (5′-CATGGGTCGATGGAATTCCGCATGCCTGCAGGTC-3′) were used. Next, the constructed minicircle parental plasmid was transformed into the ZYCY10P3S2T *E. coli* minicircle production bacterial strain (System Biosciences). A single clone was inoculated into 100 ml of Terrific Broth and grown for 12 h at 250 r.p.m. and 37 °C. Then, 0.1 % l-arabinose was added and cultured for an additional 5 h at 250 r.p.m. and 30 °C. The bacterial cells were then pelleted, and minicircles were extracted using the EndoFree Maxi Plasmid Kit (Tiagen) and verified by 1 % agarose gel.

### Transfection and infection

A total of 3×10^5^ HEK293T cells were seeded into 12-well plates 20 h prior to transfection. The next day, 0.5 µg of Cas9: gRNA expression plasmid and 0.5 µg of donor plasmid were cotransfected using jetPRIME transfection reagent (Polyplus) according to the manufacturer’s protocol. At 24 h post-transfection, the cells were infected with HSV at a m.o.i.=1. For the T7E1 assay, the cells were transfected with 1 µg of Cas9: gRNA expression plasmid alone. Viral samples were collected 48 h post-infection and then subjected to three freeze–thaw cycles. To determine the optimal conditions for NHI, different cotransfected plasmid mass ratios (mass ratio of Cas9: gRNA expression plasmid and donor plasmid at 2 : 1, 1 : 1, 1 : 2 and 1 : 4 of 1 µg plasmid in total), m.o.i. (0.01, 0.1, 1 or 10) and virus harvest times (12, 24, 36, 48, 60, 72 h after infection) were tested.

### Plaque assay and knock-in efficiency calculation

A total of 8×10^5^ Vero cells were seeded into six-well plates 18 h prior to infection. HSV was serially tenfold diluted from 10^1^ to 10^6^, and 1 ml of diluted virus was added to the cells and incubated for 2 h at 37 °C. After incubation, the viral supernatant was discarded, and MEM containing 2 % FBS and 1 % agarose was added. Then, 3 to 5 days later, the plaques became visible under invert microscope, and EGFP-positive plaques were randomly marked and counted under a fluorescence microscope (TE2000-U, Nikon) or using channel Alexa 488 of ChemiDoc imaging system (Bio-Rad). The marked EGFP-positive plaques were picked and grown in Vero cells seeded in 96-well plates 20 h prior to infection. The knock-in efficiency was calculated by calculating the ratio of the EGFP-positive plaque number and the total plaque number. The plaque diameter was measured and analysed using ImageJ 1.46 r software.

### Genome cleavage efficiency and fragment insertion detection assay

The edited viral genomes were extracted using the MiniBEST Viral RNA/DNA Extraction Kit Ver.5.0 (TaKaRa), and fragments across the cut site and foreign fragments were amplified using Q5 High-Fidelity DNA Polymerase. To detect genome cleavage efficiency using amplicon sequencing, the purified PCR products were ligated to the pMD19-T vector (TaKaRa), and amplicons were randomly picked and then subjected to Sanger sequencing to calculate the gene modification efficiency. To detect genome cleavage efficiency using T7 endonuclease I (T7E1), the purified PCR products were denatured, reannealed and digested with three units T7E1 (New England BioLabs) for 30 min at 37 °C. The digested products were analysed by 2 % agarose gel. The editing efficiency detected by restriction fragment length polymorphism was calculated by using a previously described formula [20].

### Firefly luciferase activity assay

Vero cells were infected with wild-type or recombinant Luc virus at a m.o.i.=3 for 2 h at 37 °C and washed with PBS once, following which cell lysates were collected at 24 h postinfection. The luciferase activity was analysed using firefly luciferase assay reagent (Promega) and a Fluoroskan Ascent FL (Thermofisher Scientific), and the values were multiplied by a scaling factor of 10^6^.

### Western-blot assay

In total, 10 μg of each cell lysate sample was analysed by 10 % SDS-PAGE. The proteins were transferred onto a polyvinylidene difluoride member using a Trans-Blot Turbo system (Bio-Rad, Hercules, CA, USA). The membrane was blocked for 1 h using 5 % nonfat milk in TBST buffer (50 mmol l^−1^ Tris base, 150 mmol l^−1^ NaCl, pH 7.5, 0.05 % Tween 20). The membrane was incubated with anti-GFP, anti-gB, anti-Cas9 and anti-β-actin antibodies (anti-GFP=1 : 1000, AG281, Beyotime; anti-gB=1 : 12 500, ab6506, Abcam; anti-β-actin=1 : 12 500, A1978, Sigma-Aldrich; anti-Cas9=1 : 1000, ab191468, Abcam) for 1 h after blocking. After washing five times, the membrane was incubated with HRP-conjugated goat anti-mouse antibody (1 : 10 000, Sigma-Aldrich) for 40 min and then washed three times with TBST buffer. Signals were visualized using Amersham ECL Prime Western Blotting Detection Reagent (GE Healthcare).

### End-point dilution assay

A total of 3×10^5^ Vero cells were seeded into 12-well plates 18 h prior to infection. The next day, the Vero cells were infected with recombinant HSV1-ZW6-TK-EGFP virus at a m.o.i.=1 for 2 h at 37 °C. After incubation, the viral supernatant was discarded, and MEM containing 2 % FBS with or without 100 μg ml^−1^ acyclovir was added. Then, the viruses were collected 24 h post-infection. On the third day, 1×10^4^ Vero cells were seeded into 96-well plates 18 h prior to infection. Harvested HSV was tenfold serially diluted from 10^1^ to 10^8^, and 100 µl of dilute virus was added to Vero cells and incubated with the cells at 37 °C. Three days post-infection, the numbers of CPE-positive and CPE-negative cells were recorded to calculate the TCID-50 titre according to the Reed and Muench formula [21].

### Quantitative PCR (qPCR)

The viral DNA genome and MC donor plasmid were extracted from infected cells at the indicated time points using a viral DNA kit (Omega Biotek). Nine fragments were amplified separately using corresponding sets of primers, for fragment 1 the primers are qPCR 1F (5′- CGATATGAGGAGCCAGAAC −3′) and qPCR 1R (5′- CGAGCCGATGACTTACTG −3′); for fragment 2 the primers are qPCR 2F (5′- GGGGACCGTCTATATAAACC −3′) and qPCR 2R (5′- CCCTATTGGCGTTACTATGG −3′); for fragment 3 the primers are qPCR 3F (5′- GAAGAACGGCATCAAGGT −3′) and qPCR 3R (5′- GCTCAGGTAGTGGTTGTC −3′); for fragment 4 the primers are qPCR 4 F-1 (5′- TGTCCAAACTCATCAATGTATC −3′) and qPCR 4 R-1 (5′- GATCTTGGTGGCGTGAAA −3′); for fragment 5 the primers are qPCR 5F (5′- TTCGCCAATGACAAGACG −3′) and qPCR 5R (5′- GGGCACAGGTACACTATC −3′); for fragment 6 the primers are qPCR 2F and qPCR 4 F-1; for fragment 7 the primers are qPCR 2F and qPCR 4 R-1; for fragment 8 the primers are qPCR 10 F-2 (5′- GGCGCGACTAGTGAATTG −3′) and qPCR 2R; for fragment 9 the primers are qPCR 11F (5′- GACTTCCGTGGCTTCTTG −3′) and qPCR 11R (5′- CAGGTCCACTTCGCATAT −3′); The amplification efficiency of the primer pairs was determined by calculating the relative DNA content, which was normalized to the viral genome or donor plasmid internal control using relative quantification PCR. The copy numbers of different fragments at the indicated time points were calculated by using absolute quantification PCR. qPCR was performed on a LightCycler 480 (Roche) using ChamQ SYBR Color qPCR Master Mix (Vazyme).

For RT-qPCR, the ZW6-TK-EGFP and ZW6 virus infect the 3×10^5^ Vero cells at m.o.i.=3 respectively, after 24 h, the cells were lysed in RNAiso (Takara) and total RNA were extracted by using Direct-zol RNA MiniPrep kit (Zymo Research). The total RNA was reverse transcript by using HiScript III RT SuperMix (Vazyme) and detected by ChamQ SYBR Color qPCR Master Mix (Vazyme), for TK the primers are qPCR 1F and qPCR 1R; for GAPDH (glyceraldehyde-3-phosphate dehydrogenase) the primers are qPCR-G027 (5′- AGTCAACGGATTTGTCGTA −3′) and qPCR-G028 (5′- GGGTGGAATCATACTGGAAC −3′). The relative ratio was calculated using 2^-ΔΔ^CT method.

### Statistical analysis

All data were analysed by Student's *t*-test using GraphPad PRISM 8.0 software and are presented as the mean±sd, and a *P*-value<0.05 was considered as statistically significant.

## Results

### In editing the herpes simplex virus (HSV) genome, the insertion efficiency of the NHI strategy was higher than that of the HR strategy and positively correlated with the target site cleavage efficiency

**Fig. 1.**
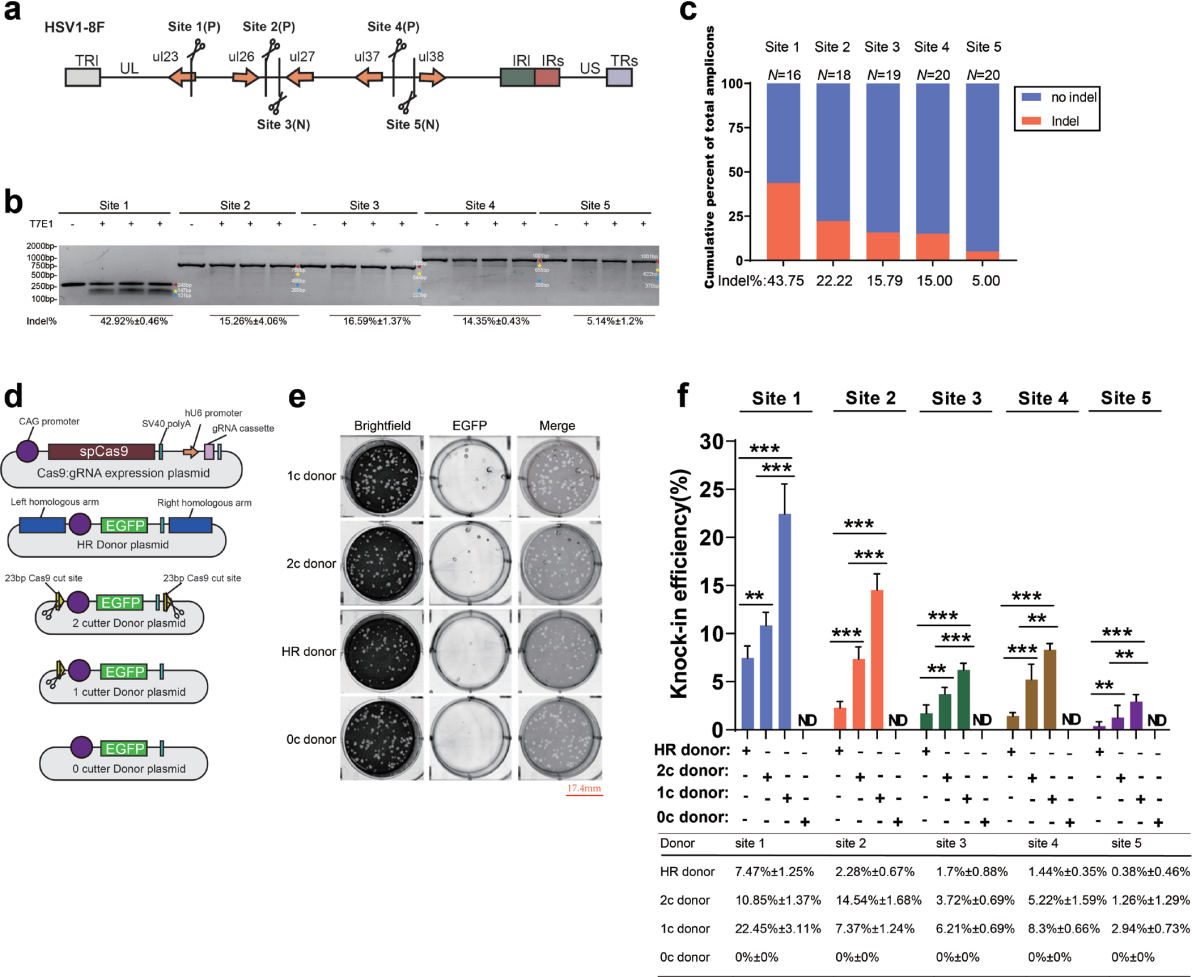
Efficiency of NHI-mediated genome editing is higher than that of HR-mediated genome editing at different sites of the HSV1 genome. (a) Schematic of five CRISPR/Cas9 target sites in the HSV1-8F genome (P, positive strand; N, negative strand). Cleavage activity at five target sites was detected by (b) using T7E1 endonuclease and (c) sequencing the amplicon of the edited HSV genome when 1 µg of Cas9: gRNA plasmid was transfected into HEK293T cells before their infection with HSV1 (m.o.i.=1) at 24 h post-transfection, with the virus harvested at 48 h post-infection. (d) Schematic of the Cas9: gRNA expression plasmid and donor plasmid. Homologous sequences (1 kb) flanked the GFP cassette used to construct the HR donor plasmid. For the NHI donor plasmid, the 2-cut donor (2 c donor) plasmid was constructed such that the EGFP cassette was flanked by the reverse complementary sequence of a 23 bp cut-site sequence, and one copy of the cut-site sequence was placed upstream of the EGFP cassette to construct the 1-cut donor (1 c donor), while the 0-cut donor (0 c donor) contained no cut sites and served as a negative control. (e) Visualization of EGFP-positive plaques generated via NHI at site 2, when 0.5 µg of Cas9: gRNA plasmid and 0.5 µg of donor plasmid were cotransfected in HEK293T cells, which were infected with HSV1 (m.o.i.=1) at 24 h post-transfection, following which viruses were harvested at 48 h post-infection. Scale bar=17.4 mm. (f) Efficiency of EGFP cassette knock-in at five target sites via NHI and HR with same condition as (d). Data were analysed by Student's *t*-test and are presented as the mean±sd from three independent experiments (**, *P*<0.01; ***, *P*<0.001; nd, no detection).

Herpes simplex virus type 1 (HSV1), which has one of the fastest replication rates among members of Herpesviridae, was selected as the research system in this study. Five target sites in the HSV1 genome with high specificity that do not affect viral replication after their mutation were selected using the CRISPOR (http://crispor.tefor.net) target-site prediction tool; sites 1, 2 and 4 were in the positive strand of the genome, while sites 3 and 5 were in the negative strand (Fig. 1a and Table 1), and the sequences are conserved between different strains [Fig. S1 (available in the online version of this article) ]. Then, 24 h after the introduction of a CRISPR/Cas9 system recognizing a specific target site into HEK293T cells, the cells were infected with HSV1 virus, and progeny viruses in the media supernatant and cells were harvested after infection for 48 h. The efficiency of different target-site cutting by CRISPR/Cas9 was evaluated by T7 endonuclease 1 mismatch assay, which showed a significant difference in the cutting efficiency of the CRISPR/Cas9 system at the different target sites. The cutting efficiency was highest at site 1 and reached 42±0.46 %. The cutting efficiencies at sites 2, 3 and 4 were similar and ranged from 15–17 %, although the site-2 cutting efficiency was slightly higher than the cutting efficiencies of the latter sites. Finally, the cutting efficiency of the CRISPR/Cas9 system at site 5 was only 5.14±1.2 % (Fig. 1b). Further evaluation by Sanger sequencing showed similar results (Fig. 1c).

**Table 1.** Information on the sgRNAs of five sites. Results were predicted using the CRISPOR tool (http://crispor.tefor.net). A higher score indicates higher possible sgRNA specificity, editing efficiency and rate of out-of-frame DNA repair

SgRNA	Genome position (HSV1-8F)	Guide sequence + *PAM*	Specificity score		sgRNA target region	sgRNA target DNA strand	Off-target site prediction
			MIT	CFD			
Site 1	47709–47728	GAGGGCGCAACGCCGTACGT *CGG*	100	100	UL23 (nonessential gene)	positive	0 off-target sites
Site 2	52847–52866	CAATGCAAAAGCCTTCGACG *TGG*	100	100	Intergenic region	positive	0 off-target sites
Site 3	52926–52907	CGTGACAAAACGGACCCCCT *TGG*	100	100	Intergenic region	negative	0 off-target sites
Site 4	84167–84186	GGCGAATTATCGATCCCCCG *CGG*	100	100	Intergenic region	positive	0 off-target sites
Site 5	84517–84498	CGCGACCTAACAAAACCCGG *GGG*	100	100	Intergenic region	negative	0 off-target sites

**Fig. 2.**
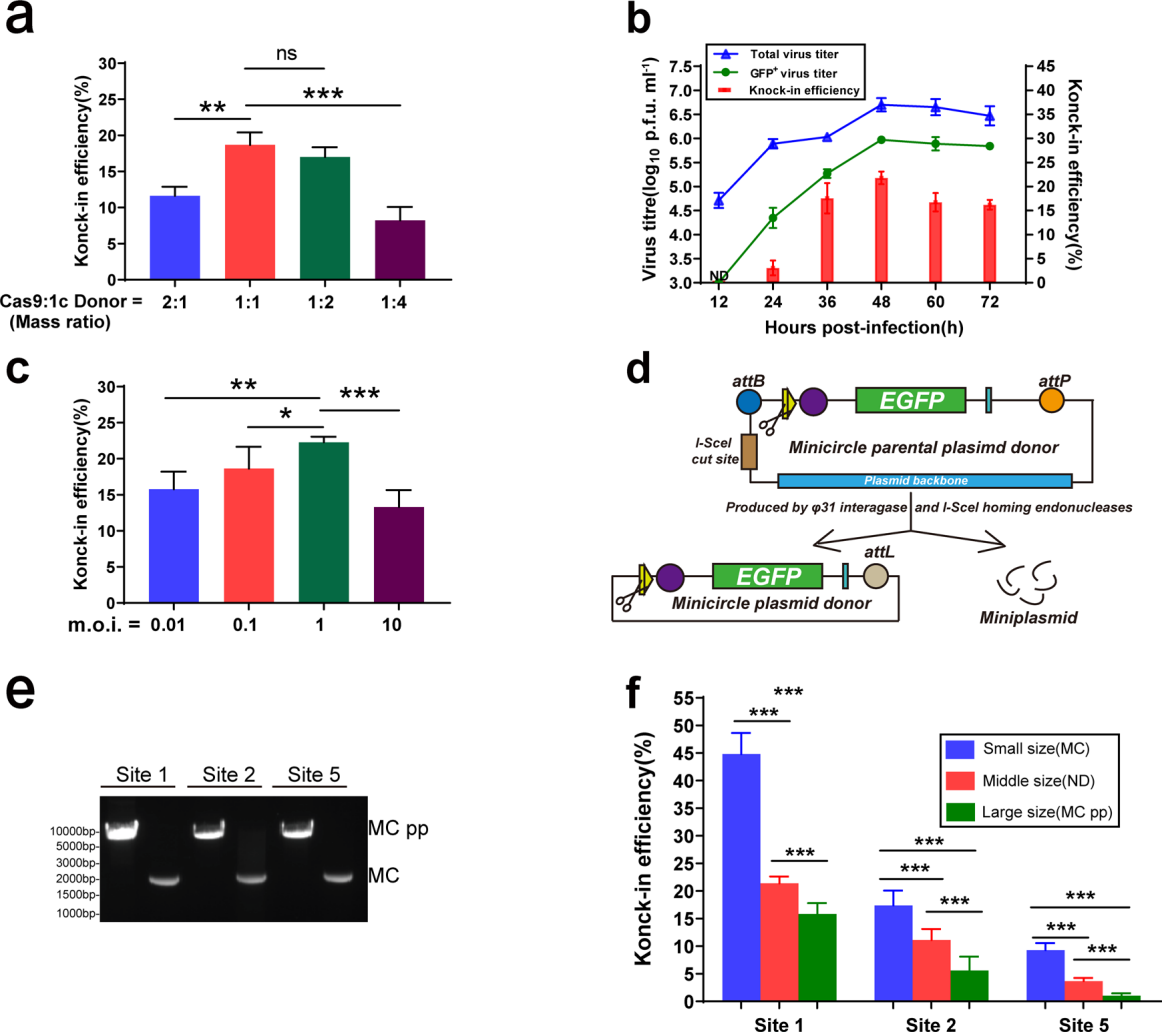
Factors affecting the knock-in efficiency with NHI. (a) Efficiency of EGFP cassette knock-in at site 1 with different cotransfection plasmid mass ratios. (b) The total or EGFP-positive viral titres and efficiency of EGFP cassette knock-in at site 1 with different virus harvest times. (c) Efficiency of EGFP cassette knock-in at site 1 with different m.o.i. (d) Schematic of the production of the minicircle plasmid. During the induction, the minicircle plasmid was produced from minicircle parental plasmid by digestion with I-SceI endonucleases and recombination with φ31 integrase. (e) Gel electrophoresis of MCpp and MC donor plasmids containing sites 1, 2 and 5, respectively. (f) Efficiency of EGFP cassette knock-in at sites 1, 2 and 5 when the MCpp, ND and MC donor plasmids, respectively, were used. Data were analysed by Student's *t*-test and are presented as the mean±sd from three independent experiments (*, *P*<0.05; **, *P*<0.01; ***, *P*<0.001; ns, not significant).

Foreign-gene donor plasmids containing the EGFP expression cassette were designed and used to compare foreign-gene insertion efficiencies at the five targets with different knock-in strategies. For the HR strategy, 1 kb sequences upstream and downstream of the CRISPR/Cas9 cleavage target were amplified and used to flank the EGFP expression cassette (Fig. 1d). For the NHI strategy (Fig. S2), a homologous CRISPR/Cas9 cleavage site was introduced upstream of the EGFP cassette, or two cleavage sites were used to flank both sides of the EGFP expression cassette in the donor plasmid (Fig. 1d). At the same time, a donor plasmid containing no homologous sequences or cleavage sites was used as a control. To avoid recleavage of the viral genome by Cas9 after insertion of the foreign gene, the donor plasmid used with HR and NHI was resistant to CRISPR/Cas9 when the foreign fragment was inserted. The CRISPR/Cas9 system recognizing a target site was introduced into HEK293T cells together with the donor plasmid, and the cells were then infected with HSV1 virus. After infection for 48 h, progeny viruses were harvested. Using a plaque assay to detect recombinant progeny viruses after serial dilution, a large number of recombinant progeny viruses exhibited green fluorescence (Fig. 1e). The insertion efficiency with the donor plasmid containing one cleavage site (Fig. 1f and 1 c donor) was twofold higher than that with the donor plasmid containing two cleavage sites (Fig. 1f and 2 c donor). Moreover, among all target sites tested, the efficiency of the NHI strategy was significantly higher than that of the HR strategy, especially for site 5, at which the recombination efficiency was lowest. The NHI strategy was 16-fold more efficient than the HR strategy. In addition, when the same recombination strategy was utilized, foreign-gene knock-in into the viral genome was more efficient at target sites with a higher cleavage efficiency, suggesting that the foreign gene knock-in efficiency and efficiency of cleavage at the target site are positively correlated.

### Factors that determine the insertion efficiency

To further increase the efficiency of foreign-gene insertion into the viral genome using the NHI strategy, we explored the factors that influence insertion efficiency.

First, we compared the effect of the transfected Cas9 plasmid and donor plasmid mass ratio on the insertion efficiency, with the total mass of the cotransfected plasmids controlled. The cotransfected plasmid mass ratio was adjusted, and when the mass ratio of Cas9 plasmid to donor plasmid was 1 : 1, the highest knock-in efficiency, which reached approximately 19%, was achieved (Fig. 2a).

Subsequently, viruses were harvested at different time points after infection, and the infectious progeny viral titre was detected. Twelve hours after viral infection, no progeny virus expressing EGFP was detected, and a small proportion of viruses expressing EGFP were detected at 24 h post-infection. The proportion of recombinant viruses gradually increased with increasing infection and replication time, and the proportion of recombinant viruses expressing green fluorescent protein peaked after infection for 48 h, following which the proportion of recombinant virus expressing green fluorescent protein declined (Fig. 2b).

Finally, we explored the effect of viral m.o.i. during infection on knock-in efficiency. The proportion of recombinant viruses carrying foreign fragments was slightly different with different m.o.i. (Fig. 2c). In this study, the proportion of recombinant viruses expressing the target protein was highest when the m.o.i.=1.

In addition, when foreign-gene knock-in was previously carried out with the HR strategy, the knock-in efficiency was inversely associated with the length of the knock-in fragment (unpublished data). Therefore, an experiment to further compare the effect of the length of the foreign gene on the insertion efficiency with the NHI strategy was carried out. The plasmid backbone was efficiently removed by homing enzyme digestion and φ31 integrase ligation (Fig. 2d), following which a minicircle plasmid containing only the gene-expression cassette was constructed [22]. Here, we compared the effects of three target sites with different insertion efficiencies (sites 1, 2 and 5) and three DNA plasmids with different sizes (Fig. 2e) containing one CRISPR/Cas9 target site: the minicircle parental plasmid (MCpp, 5738 bp), which contained the redundant plasmid backbone; normal sized 1-cut donor plasmid (ND, 4303 bp); and smallest minicircle plasmid (MC, 1740 bp). The insertion efficiencies with different sized donor plasmids were significantly different (Table 2). The insertion efficiency at the three sites consistently showed that the smaller the foreign fragment was, the higher the insertion efficiency was, with the highest efficiency of 45 % obtained using the minicircle donor with insertion at site 1. The insertion efficiency at site 5, which had the lowest insertion efficiency, was 10 % with the minicircle donor plasmid (Fig. 2f).

**Table 2.** Summary of knock-in efficiency of NHI strategy using the different length of donor plasmid. Data are presented as the mean±sd of virus plaque number or knock-in efficiency from three independent experiments

Donor	Site 1				Site 2		Site 5		
	GFP^+^ plaque	Total plaque	knock-in efficiency	GFP^+^ plaque	Total plaque	knock-in efficiency	GFP^+^ plaque	Total plaque	knock-in efficiency
MC	14.8±3.26	32.93±6.32	44.8±3.85 %	26.66±9.64	163.44±48.43	17.41±2.68 %	17.22±2.16	188.66±11.55	9.29±1.27 %
ND	9.08±0.8	41.2±1.59	21.39±1.25 %	19.11±6.19	171±41.62	11.13±1.98 %	6.11±1.05	165.66±5.38	3.68±0.58 %
MC pp	7±1.24	44.06±5.67	15.86±1.95 %	5.66±3.77	149.77±49.01	5.61±2.51 %	1.66±0.7	156.88±17.15	1.05±0.43 %

Consequently, by optimizing the mass ratio of the cotransfected plasmids, the m.o.i., and the virus harvest time and shortening the inserted foreign gene, the efficiency of foreign-gene insertion into the HSV genome was increased.

### Foreign genes can be efficiently inserted into a low-passage clinical virus and remain stable

**Fig. 3.**
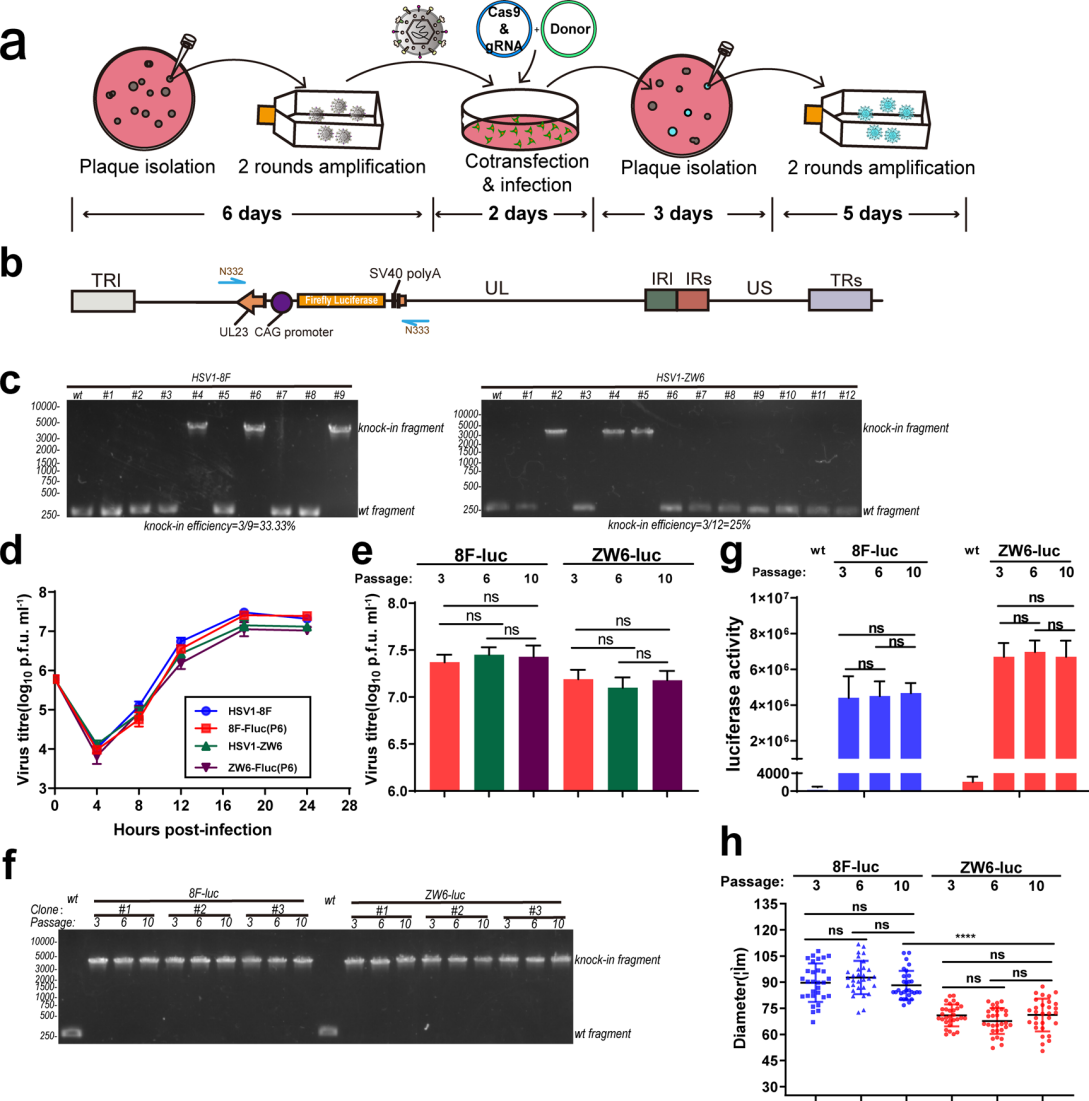
Rapid construction of stable passaged recombinant reporter virus from freshly isolated clinical HSV1 via NHI. (a) Schematic diagram of the rapid construction of recombinant virus from clinical isolates using the NHI strategy within 3 weeks. (b) Schematic of the HSV1 genome in which the firefly luciferase cassette has been integrated at site 1 via NHI. The half arrow in blue indicates the amplification and sequencing primers. (c) Detection of firefly luciferase cassette knock-in efficiency in different HSV1 strains by PCR. Viral plaques in one well were picked randomly, and genomes were extracted for PCR detection after infection for 2 days, with the wild-type virus serving as a negative control (HSV1-8F is a laboratory passage strain, while HSV1-ZW6 is a clinical isolate). (d) Comparison of the growth characteristics of recombinant HSV1 (P6) and wild-type HSV1 after recombinant HSV1 had been serially passaged on Vero cells ten times using viral one-step growth curves. (e) The titres of different strains of recombinant HSV1 at the indicated passage number. (f) PCR detection of the fragment integrated in three recombinant virus clones with different HSV1 strain genomes, with the wild-type virus serving as a negative control. (g) Firefly luciferase activity of different recombinant HSV1 strains at the indicated passage number after infection at m.o.i.=3 for 24 h, with the wild-type virus serving as a negative control. (h) Measurement of the plaque diameters of different recombinant HSV1 strains at the indicated passage number after infection for 60 h (*n*=30; ****, *P*<0.0001; ns, not significant). Data were analysed by Student's *t*-test and are presented as the mean±sd, and the data in (e) and (g) were from three independent experiments.

A highly efficient foreign-gene insertion strategy means that only a small amount of virus and less time and work are needed to effectively obtain the recombinant virus. We isolated clinical HSV1 virus from the blister fluid of a labial herpes patient, and after one round of plaque purification and two rounds of expansion in Vero cells, the low-passage clinical ZW6 virus strain was obtained and subsequently sequenced (the whole-genome sequence: GenBank KX424525.1). Compared to the laboratory cell-adapted HSV1 strain (HSV1-8F), the ZW6 strain has lower titres and smaller plaques. We used a small amount of virus (3×10^5^ p.f.u., m.o.i.=1) and integrated the firefly luciferase (Luc) expression cassette into the viral genome at site 1 using the NHI strategy (Fig. 3a, b). After a plaque assay with the harvested virus, the progeny virus was randomly picked, and PCR was performed to detect the Luc gene insertion efficiency. The firefly luciferase expression cassette was integrated into the 8F and ZW6 HSV1 strains with a 33.3 and 25% knock-in efficiency, respectively (Fig. 3c). Because plaques exhibited a dispersed distribution on the cell-culture well, purified recombinant HSV could be obtained after one round of plaque purification, and no wild-type virus contamination was detected using PCR. To detect the stability of the inserted fragment, HSV 8F-Fluc and ZW6-Fluc viruses were serially passaged under the same passage conditions, and the third-, sixth- and tenth-generation viruses were then selected for the assay. First, the growth properties of the P6 recombinant virus were assayed by its one-step growth curve, which showed that there was no significant difference in the one-step growth curve between the Luc-expressing and wild-type viruses (Fig. 3d). Over ten consecutive passages, the titre of HSV-Luc recombinant viruses harvested at 24 h post-infection was not significantly different between each passage (Fig. 3e), and the titre of the cell-adapted 8F strain was higher than that of the low-passage clinical ZW6 strain. By PCR detection of the region outside of the insertion site, no fragment deletion or lengthening was found in the region containing the inserted fragment during serial passage (Fig. 3f). After infection with the same amount of different HSV1-Luc virus for 24 h, the luciferase activity of the cell lysate was detected, and the luciferase activity signal was stable between different passage numbers. Unexpectedly, the firefly luciferase catalytic activity of the ZW6-Luc virus was slightly higher than that of the 8F-Luc virus (Fig. 3g). Moreover, 3 days after recombinant virus infection, 30 plaques were randomly selected for plaque-size determination, which showed that the ZW6 virus plaque size was significantly smaller than the 8F virus plaque size after infection on Vero cells. Plaques formed by the 8F-Luc and ZW-Luc strains in different passages numbers were compared, and both viruses remained stable (Fig. 3h).

High-efficiency gene insertion with the NHI strategy enables the rapid insertion of foreign genes and maintains the genetic stability in both laboratory and clinical isolates, significantly enhancing the efficiency of recombinant virus construction and maintaining the clinical features of the recombinant virus.

### Fidelity of NHI for the insertion of foreign genes into the HSV genome varies depending on the sequence background of the cleavage site

**Fig. 4.**
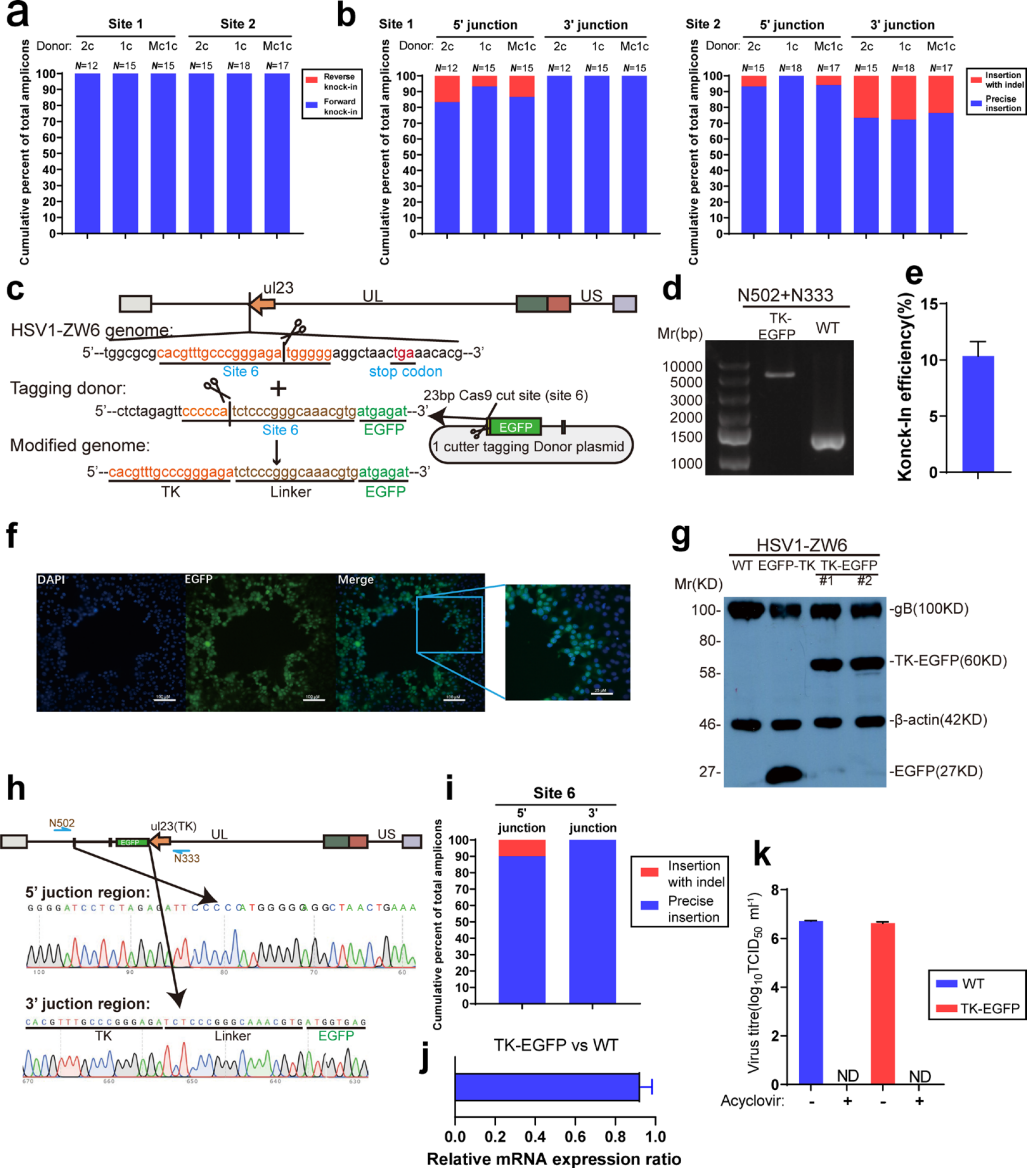
Accurate and directional insertion of a foreign gene into the HSV genome via NHI. (a, b) Analysis of the direction and accuracy of EGFP cassette insertion at sites 1 and 2 with NHI by sequencing the upstream and downstream junction sequences of randomly picked EGFP-positive viruses constructed with 2 c, 1 c and MC donor plasmids. (c) Schematic of the fusion of the EGFP gene to the C-terminus of the HSV1-ZW6 TK gene via NHI. The reverse complementary sequence of the 23 bp cut-site sequence (site 6) was placed upstream of the EGFP sequence to construct the donor plasmid, and 17 bps of the cut site served as a linker sequence after insertion. (d) Detection of EGFP fused to TK in HSV1-ZW6 strains by PCR. GFP^+^ virus plaques were picked, the genomes were extracted for PCR detection after infection for 2 days, with the wild-type virus serving as a negative control, and (e) the efficiency of EGFP fused with TK gene was calculated. (f) Microscopic image of TK-EGFP-positive plaques after infection for 3 days. (g) Western-blot detection of the expression of the TK-EGFP fusion protein after infection with two recombinant TK-EGFP virus clones for 24 h at an m.o.i.=3 (EGFP-TK is a recombinant virus in which the EGFP expression cassette has been integrated into site 1 that served as a negative control, and glycoprotein B (gB) served as a viral infection control). (h), (i) Sequencing analysis of accuracy of the EGFP gene fused with the TK gene (*n*=10). The half arrow in blue indicates the amplification and sequencing primers. (j) The mRNA expression level of TK in TK-EGFP virus was compared to the wild-type virus, total RNA was extracted 24 h after the TK-EGFP virus infected the Vero cells at m.o.i.=3, then the expression level of TK was detected by RT-qPCR, and compared to TK of ZW6 (the levels of TK mRNA were normalized to GAPDH mRNA and shown as a relative ratio). (k) To determine the TK activity of recombinant TK-EGFP virus, the virus was harvested in the absence or presence of 100 µg ml^−1^ acyclovir at 24 h post-infection. Then, the titre was detected by a limiting dilution assay, and wild-type HSV1-ZW6 served as a positive control. Data were analysed by Student's *t*-test and are presented as the mean±sd from three independent experiments. (ND, no detection).

The factors that affect the fidelity of NHEJ to repair the cellular genome remain unclear; therefore, repair outcomes cannot yet be predicted. The fidelity of the NHEJ strategy to repair episomal viral genomes also remains to be assessed. When NHEJ was previously used to knockout the HSV gene, the fact that the precisely repaired genome could be recleaved by CRISPR/Cas9 until a resistant indel appeared was ignored; thus, the error proneness of NHEJ was amplified. Due to the design of NHI, in which the CRISPR/Cas9 recognition site is cleaved after the foreign fragment is integrated, recutting can be avoided; hence, the fidelity of NHEJ can be more accurately determined. Hence, we randomly picked HSV1-EGFP recombinant progeny virus in which the EGFP cassette had been inserted at sites 1 and 2 using different kinds of donor plasmids, and the sequences at both the upstream and downstream junctions were detected by PCR and Sanger sequencing. First, the sequencing results showed that among all the picked recombinant viruses, the foreign genes had been integrated in a forward direction, and no reverse insertion was detected (Fig. 4a), indicating that NHI is a directional gene knock-in strategy. Second, at the same target site, regardless of whether the normal plasmid (1 c or 2 c plasmid) or the minicircle plasmid had been used as the donor plasmid, the fidelity of the junction sequence was similar. At site 1, the proportion of genes precisely inserted at the upstream junction was above 83%, and that at the downstream junction was 100 % (Fig. 4b). The proportion of genes precisely inserted at the upstream junction of site 2 was close to 100%, but only 72.22 % of the inserted genes had been inserted precisely at the downstream junction (Fig. 4b). These results suggest that the fidelity of NHEJ varies with different sequence backgrounds. Furthermore, we compared the sequencing results with the predicted repair results from the CRISPR/Cas9 DNA cleavage double-stranded DNA repair outcome prediction tool Favored Outcome of Repair Events of Cas9 Target (FORECasT). The type of mutation in the recombinant virus constructed using NHI was similar to the predicted result, which indicates that the type of mutation that occurs using NHI can be predicted (data not shown).

Although the fidelity of the NHI strategy to mediate foreign-gene insertion into the HSV genome is between 70–100 %, due to its high insertion efficiency, the NHI strategy can still be used to tag viral proteins with a fluorescent protein. Hence, we selected a gRNA that facilitates Cas9-mediated cleavage at eight nucleotides before the stop codon of the thymidine kinase (TK) gene in HSV1-ZW6 and designed a one-cut EGFP tagging donor plasmid such that when the donor plasmid is cleaved, the remaining 17 bp gRNA sequence will become the linker sequence between TK and EGFP (Fig. 4c). Approximately 10.33 % of the HSV1-ZW6 viruses were labelled with EGFP following NHI (Fig. 4d). Since the TK protein of HSV1 can enter the nucleus [23], all the green fluorescent proteins were observed in the nucleus under a fluorescence microscope, indicating that the TK-EGFP fusion protein was expressed (Fig. 4f). After a round of plaque purification, Western blotting (Fig. 4g) and sequencing (Fig. 4h) were performed, and the results confirmed that the EGFP protein was labelled with TK protein, moreover, only one clone showed indel in ten sequenced clones (Fig. 4i). Besides, the expression of TK in TK-EGFP virus show 0.927±0.056 folds compare to wild-type virus (Fig. 4j), indicates the fusion of EGFP barely affect the expression level of TK. Furthermore, when 100 mg ml^−1^ acyclovir was added to the medium after infection, the recombinant virus could not produce progeny virus, indicating that the TK-EGFP fusion protein retained its enzymatic activity (Fig. 4k). Therefore, the NHI strategy can also be used to efficiently label HSV viral proteins.

### Dynamic process of NHI-mediated foreign-gene insertion into the HSV1 genome

The viral genome replicates rapidly and then produces copious target sites, so the process from viral infection to the generation of recombinant progeny virus is rapid and relatively complex compared to gene insertion in the cellular genome. To comprehend this process, we assessed changes to the pivotal fragment of the entire gene-insertion process using quantitative pulse analysis.

**Fig. 5.**
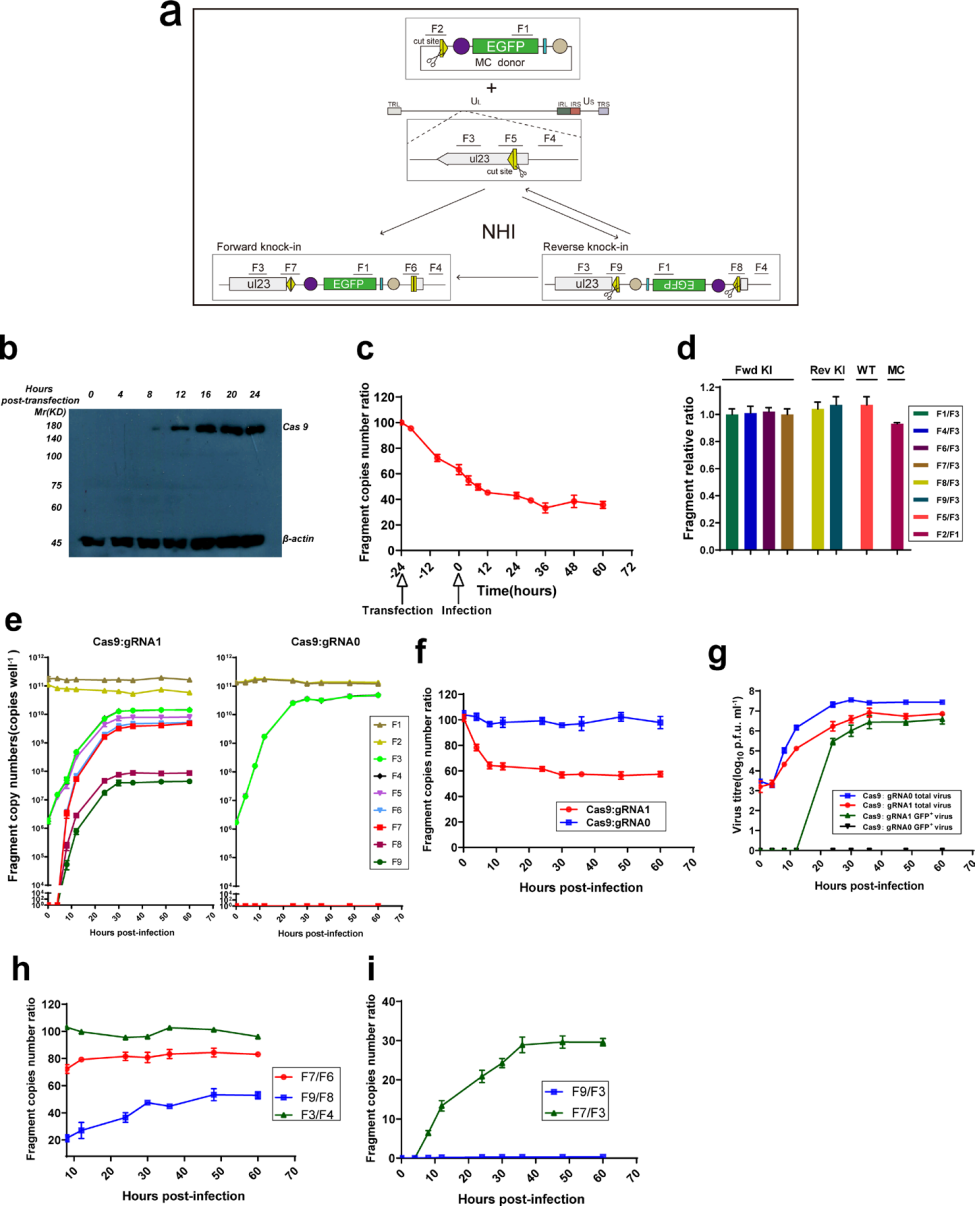
The dynamic process of gene knock-in via NHI into the HSV genome. (a) Schematic of EGFP cassette insertion at site 1 via NHI. Both the MC donor plasmid and HSV genome could be cleaved simultaneously by the Cas9:gRNA1 complex, and the linearized donor was then integrated into the cleaved HSV genome at site 1 in two directions via NHI. Fragments one to nine (F1 to F9) were designed to reflect the dynamic NHI process in HSV. (b) Western blotting was performed to detect the time course of Cas9 protein expression when 0.5 µg of Cas9:gRNA1 and 0.5 µg of MC donor plasmid were cotransfected into HEK293T cells. (c) The percentage of MC donor plasmid cleavage at the indicated time point, with the MC donor plasmid extracted at the indicated time point and F2 quantified using qPCR. F1 served as an internal control used to calculate the ratio. (d)The amplification efficiencies of the primer pairs were detected by relative qPCR. Fragments 1, 4, 6 and 7 were amplified from the purified EGFP cassette forward knock-in (Fwd KI) HSV genome, fragments 8 and 9 were amplified from the purified EGFP cassette reverse knock-in (Rev KI) HSV genome, fragment 5 was amplified from the wild-type (WT) HSV genome, and fragment 3 served as a viral genome internal control in the above group. Fragment 2 was amplified from the MC donor plasmid, and fragment 1 served as a donor plasmid internal control. (e) The copy numbers of the different fragment in whole cell culture wells at the indicated time point with the Cas9:gRNA0 plasmid, which did not target the HSV genome, serving as a negative control. (f) The percentage of HSV genome cleavage at the indicated time points, with the HSV genome extracted at the indicated time points and F5 quantified using qPCR. F3 served as an internal control used to calculate the ratio. (g) The titres of total and GFP-positive viruses harvested at the indicated time point. (h) The ratio of the copy numbers of fragments that represent upstream and downstream junctions in forward or reverse knock-in insertion events. F7, F6, F9 and F8 were quantified using qPCR and used to calculate the ratio of upstream and downstream junctions in forward or reverse knock-in insertion events, respectively. F3 and F4 served as a pair of internal controls. (i) The ratio of the forward knock-in fragment and reverse knock-in fragment copy numbers. F9 and F7 were quantified using qPCR to reflect forward and reverse knock-in events, respectively. F3 served as an internal control used to calculate the ratio. All data were analysed by Student's *t*-test and are presented as the mean±sd from three independent experiments.

Here, we selected site 1 for the evaluation, and Cas9:gRNA1 and minicircle donor plasmid containing one cut site were cotransfected into HEK293T cells (Fig. 5a). Meanwhile, Cas9:gRNA0 plasmid that did not target a specific site was used as a control. Cells were infected with HSV1-8F (m.o.i.=10) after transfection for 24 h, and samples were collected at different time points for detection.

Before viral infection, Cas9 protein expression gradually increased after cotransfection, reaching a maximum at approximately 20 h post-transfection (Fig. 5b). Then, the abundance of F1 and F2 on the donor plasmid was detected by qPCR, with F1 representing the EGFP gene, and F2 representing the Cas9:gRNA1 cleavage site in the donor plasmid. From the copy number ratio of F2 to F1 (Fig. 5c), we observed that F2 was cleaved by the Cas9 protein at 4 h after transfection and that even if DNA repair mechanisms were active, the cleavage was efficient (cutting rate=196 copies cell min^−1^). Then, the cutting rate gradually slowed from 12 h after virus infection and reached the limit at which the donor plasmid could no longer be cut at 60 h after transfection. Finally, approximately 36 % of F2 was resistant to cleavage mediated by the Cas9 protein, which may be related to inaccurate repair by NHEJ.

After virus infection, the abundance of F3 and F4 on the HSV1 genome was quantified by qPCR, with F3 representing the HSV1 genome, and F5 representing the uncleaved cleavage sites on the HSV1 genome. Meanwhile, another fragment in the adjacent region (F4) was used as an internal control to eliminate qPCR systematic error. Additionally, interference due to the different amplification efficiencies observed with different primers was excluded, and the results from a preliminary experiment showed that the amplification efficiencies of each fragment were approximately identical when the same amount of template was introduced (Fig. 5d).

To detect the insertion course, the abundance of the junction fragments downstream (F6) or upstream (F7) of the EGFP cassette was quantified when the cassette was forward inserted into the HSV1 genome (Fig. 5a), which was designed to represent a forward knock-in event. Meanwhile, other primers were designed to detect the abundance of both junction fragments (F8, F9) after reverse insertion (Fig. 5a). The abundance of each fragment over time is shown in Fig. 5e. The level of the fragment representing the donor plasmid (=6.03×10^5^ copies cell^−1^, 0 h of infection) was significantly higher than the level of the fragment representing the viral genome (=6 copies cell^−1^, 0 h infection). Additionally, the abundance of the donor DNA remained unchanged during the insertion process, but the viral genome copy number increased during the infection process.

Moreover, the results showed (Fig. 5f) that unlike cleavage of the control (Cas9:gRNA0), the viral genome was efficiently cleaved by the Cas9 protein in the first 12 h of infection (=0.74 copies cell min^−1^), with the cutting rate gradually declining before finally reaching a plateau when approximately 45 % of the F5 was retained in the viral genome. Due to cleavage by the Cas9:gRNA1 system, the replication rate of the viral genome (Fig. 5e) and viral titre (Fig. 5g) were significantly inhibited compared to those of controls, which increased the proportion of recombinant progeny virus.

When the EGFP cassette is integrated in the forward direction, the junction sequences (upstream junction: F7, downstream junction: F6) will not be cleaved by Cas9:gRNA1 as the cut site will be disrupted, but if the EGFP cassette is integrated into the genome in the reverse direction, both junction sequences following reverse insertion (upstream junction: F9, downstream junction: F8) will be cut by the Cas9:gRNA1 system unless an indel is generated. To our surprise, when the EGFP cassette was integrated into the genome, the ligation efficiencies of the two junctions were not the same. With forward insertion, the abundance of F7 was maintained at 1.2-fold higher than that of F6 (Fig. 5h). With reverse insertion, the abundance of F9 was maintained at 2.5-fold higher than that of F8. The ligation efficiency was significantly unbalanced, but the abundance of F3 and F4, which are outside of both junctions, was always consistent.

After virus infection, the recombinant viral genome was not detected in the Cas9:gRNA0 group over the whole time course of the experiment (Fig. 5e, right), whereas in the Cas9:gRNA1 group, a small proportion of the recombinant virus genome carrying the foreign fragment was detected at 8 h after infection, which suggested that the DNA repair rate is much lower than the Cas9 cutting rate. Notably, infectious progeny virus exhibiting green fluorescence was detected at only 24 h post-infection, suggesting that a period of time is required for recombination and replication of the viral genome, which is then packaged into the progeny virus. In addition, the forward-integrated recombinant viral genome could expand rapidly 8 h after infection, whereas the reverse-integrated recombinant viral genome was always inhibited. Compared to the entire viral genome, the level of the fragment representing the reverse-integrated genome was consistently decreased after its slight increase, and the proportion of this fragment was maintained at below 0.3 % from the beginning of detection, while the proportion of fragment F7, which indicated forward insertion, increased from 1.91–29 % (Fig. 5i). Moreover, the ratio of the forward and reverse insertion fragment copy numbers reached 100 : 1 at 30 h after infection, which explains why the recombinant virus was always picked during plaque purification.

Our results on the dynamic process of NHI-mediated foreign-fragment insertion into the HSV genome reflect selection pressure exerted by the NHI strategy on nondirectional integrated viral genomes, which ensures efficient directional insertion.

## Discussion

The application of a single viral vector to support the symphony of multigene co-expression in the host cell is in demand, although ambitious and difficult to carry out efficiently. Previous work using the CRISPR/Cas9 system to edit the viral genome significantly increased the efficiency of foreign gene insertion into large DNA viral genomes, such that of HR, which is a well-established and widely used strategy. Moreover, other efficient strategies are also needed to reduce the workload in the process of selection and purification of the recombinant virus. The results of this study consistently show that compared to the HR strategy, the NHI strategy significantly improves the efficiency of foreign-fragment insertion into the HSV genome at multiple sites. In addition, donor plasmid construction was simpler with NHI than with HR, and a homologous genomic sequence was not required on the NHI donor plasmid. With NHI, only the Cas9 cleavage site needs to be added to both sides or one side of the foreign gene of interest, which greatly reduces the work required to construct the donor plasmid. For long foreign genes, the insertion of foreign gene of 6000 bp, can be inserted with 16 % efficiency. Dynamic analysis of NHI showed that the NHI strategy efficiently shortened the time required to generate the recombinant viral genome; thereafter, more infectious progeny viruses could be packaged with NHI than with HR because of rapid expansion of the recombinant viral genome. Furthermore, by cutting the viral genome with the CRISPR/Cas9 system, the proportion of viruses resistant to cleavage in which the foreign gene had not been integrated, which usually accumulate during the cut-and-repair cycle, was decreased; hence, the high recombination and repair efficiency of NHI can significantly increase the proportion of recombinant progeny virus. In this study, the insertion efficiency was close to 50 % after condition optimization, and only a small amount of low-passage clinical virus and less work were needed to quickly construct and purify recombinant viruses carrying foreign genes to avoid cell-adapted mutations that gradually accumulate as viruses are passaged.

As the CRISPR/Cas9 system is one of the immune mechanisms against prokaryotic pathogens [24], the cleavage efficiency of the CRISPR/Cas9 system needs to be superior to the phage replication efficiency in prokaryotic cells to resist invasion; as a consequence, the CRISPR/Cas9 cleavage rate is usually fast. However, the DNA repair system in eukaryotic cells inversely promotes the production of recombinant and mutant viruses, which may be one of the reasons why eukaryotic cells do not use the CRISPR/Cas9 system against pathogens. Unlike gene editing of the cellular genome, use of the CRISPR/Cas9 system during the construction of recombinant viruses in eukaryotic cells significantly inhibits wild-type virus replication [14, 25], which indirectly leads to an increase in the proportion of recombinant viruses among progeny viruses. Previously, we demonstrated that the CRISPR/Cas9 system cleaves the viral genome by precisely recognizing the target site; thus, we further utilized this specific inhibitory effect of CRISPR/Cas9 and rationally designed an insertion sequence to exert a sustained inhibitory effect on undesired sequence integration into the viral genome, which promoted a selective increase in the efficiency of fragment insertion in a particular direction (Fig. 5a). To the best of our knowledge, this study is the first to report the application of this technique to the construction of large recombinant DNA viruses.

During the optimization process, a variety of factors were found to influence the efficiency of recombinant virus production. The most critical factors were the target site cleavage efficiency and the size of the foreign fragment. The insertion efficiency and cleavage efficiency of the target site were significantly positively correlated, but there was a significant negative correlation of the insertion efficiency with the size of the foreign fragment.

The fidelity of NHEJ applied to cellular genome repair remains controversial [26, 27]. In this study, the forward-integrated junction sequence was no longer recognized by the CRISPR/Cas9 system; thus, the junction sequence error rate more accurately reflected the fidelity of NHEJ. The results of sequencing randomly picked recombinant progeny virus with different sequence backgrounds showed that the fidelity of NHEJ ranged from 70–100 % and that the fidelity of NHEJ at different sites was basically maintained at the same level after repeated trials. After analysing the repair outcomes of Cas9-induced DSBs in eukaryotic cells, Brinkman *et al*. revealed that although there was a difference in the repair outcome between sites, the Cas9 cutting rate was much greater than the rate of precise repair, which indicates that DNA repair is a relatively slow process. This finding is consistent with the results we obtained using NHI-mediated fragment insertion in the viral genome. However, inconsistent with our results, when the fidelity of DNA repair was evaluated, DNA repair was found to be an error-prone process, with approximately 73 % of the repair outcomes being imprecise. We think that the accumulation of an indel might be due to iterative cut-and-repair cycles. Our results show that the abundance of the reverse insertion fragment continued to decrease during the infection process, while the proportion of the forward insertion fragment, which could not be recut, consistently increased and the accurate insertion ratio remained at least 70 %. These findings suggest that NHEJ is a relatively accurate DNA repair mechanism and that the indel produced after repair may be a strategy to escape cutting pressure exerted by CRISPR/Cas9. Recently, several studies showed that the repair outcomes of Cas9-induced DSBs were related to the sequence composition of broken DNA ends and could be predicted based on this relationship [28, 29]. Felicity *et al*. used high-throughput sequencing to analyse Cas9-induced DSB repair outcomes and developed a model with which to predict the indel type in Cas9-induced DSB repair outcomes [29]. In our study, the indel type in the recombinant viral genome could also be predicted by this method (unpublished data). However, a method to predict the indel rates of different repair outcomes has not yet been reported. Because of insufficient data, we have not been able to summarize the factors that influence differences in indel rates at between different target sites. In addition, by analysing the dynamic NHI process, we also observed that the repair efficiency at both junctions was inconsistent, even though the target sites in junction sequences recognized by the same gRNA were significantly different in their repair efficiency (Fig. 5a), suggesting that the NHEJ repair efficiency is related to the sequence background. Several studies have shown that during the process of double-stranded DNA cleavage, Cas9 will tightly associate at one or both ends of the DSB and that the process of dissociation is slow [27, 30]. On the one hand, this slow dissociation affects correct DNA digestion by Cas9 [27]; on the other hand, slow dissociation may impair DSB repair processes [30]. In addition, the chromatin environment of the DNA ends may also affect the recruitment of DNA repair factors, which may lead to different repair efficiencies [31]. Although there is a need to optimize the fidelity of NHEJ in eukaryotic cells, due to the 70–100 % fidelity of NHEJ combined with the optimized high efficiency of NHI and because a virus such as HSV is easy to expand and purify in cell culture, it is possible to efficiently label viral proteins with tags such as EGFP, even if the fidelity of NHEJ is not high.

Consequently, use of the NHEJ strategy and rational design of the donor sequence can allow the efficient and convenient insertion of a large foreign fragment into a large DNA viral genome and will provide an effective method for the development of large DNA viral vectors.

## Supplementary Data

Supplementary material 1
