# Supplementary material for: High-efficiency nonhomologous insertion of a foreign gene into the herpes simplex virus genome
Source: J Gen Virol. 2020 Jun 30;101(9):982–96. doi: 10.1099/jgv.0.001451 (PMC7654747; doi:10.1099/jgv.0.001451)
Supplement: Supplementary material 1 [file jgv-101-982-s001.pdf]

Strain	Site 1
8F	GAGGGCGCAACGCCGTACGTCGG
17	GAGGGCGCAACGCCGTACGTCGG
ZW6A	GAGGGCGCAACGCCGTACGTCGG
KOS	GAGGGCGCAACGCCGTACGTCGG
McKrae	GAGGGCGCAACGCCGTACGTCGG
F	GAGGGCGCAACGCCGTACGTCGG

Strain	Site 2
8F	CAATGCAAAAGCCTTCGACGTGG
17	CAATGCAAAAGCCTTCGACGTGG
ZW6A	CAATACAAAAGCCTTCGACGTGG
KOS	CAATGCAAAAGCCTTTGACGTGG
McKrae	CAATGCAAAAGCCTTCGACGTGG
F	CAATGCAAAAGCCTTCGACGTGG

Strain	Site 3
8F	CGTGACAAAACGGACCCCCTTGG
17	CGTGACAAAACGGACCCCCTTGG
ZW6A	CGTGACAAAACTGACCCCCTTGG
KOS	CGTGACAAAACGGACCCCCTTGG
McKrae	CGTGACAAAACGGACCCCCTTGG
F	CGTGACAAAACGGACCCCCTTGG

Strain	Site 4
8F	GGCGAATTATCGATCCCCCGCGG
17	GGCGAATTATCGATCCCCCGCGG
ZW6A	GGCGAATTATCGATCCCCCGCGG
KOS	GGCGAATTATCGATCCCCCGCGG
McKrae	GGCGAATTATCGATCCCCCGCGG
F	GGCGTATTATCGATCCCCCGCGG

Strain	Site 5
8F	CGCGACCTAACAAAACCCGGGGG
17	CGCGACCTAGCAAGACCCGGGGG
ZW6A	CGCGACCTAGCAAGACCCGGGGG
KOS	CGCGACCTAGCAAGACCCGGGGG
McKrae	CGCGACCTAACAAAACCCGGGGG
F	CGCGACCTAGCAAGACCCGGGGG

Figure S1. Information on variability and conservation of the five target sites between several strains of HSV-1. The five target sites of HSV-1 strain 17(GenBank: NC_001806), ZW6A (GenBank: KX424525.1), KOS (GenBank: JQ673480), Mckrae (GenBank: JQ730035), F (GenBank: GU734771) were aligned with strain 8F. The characters in red indicate the diversity of nucleotide in these sites.

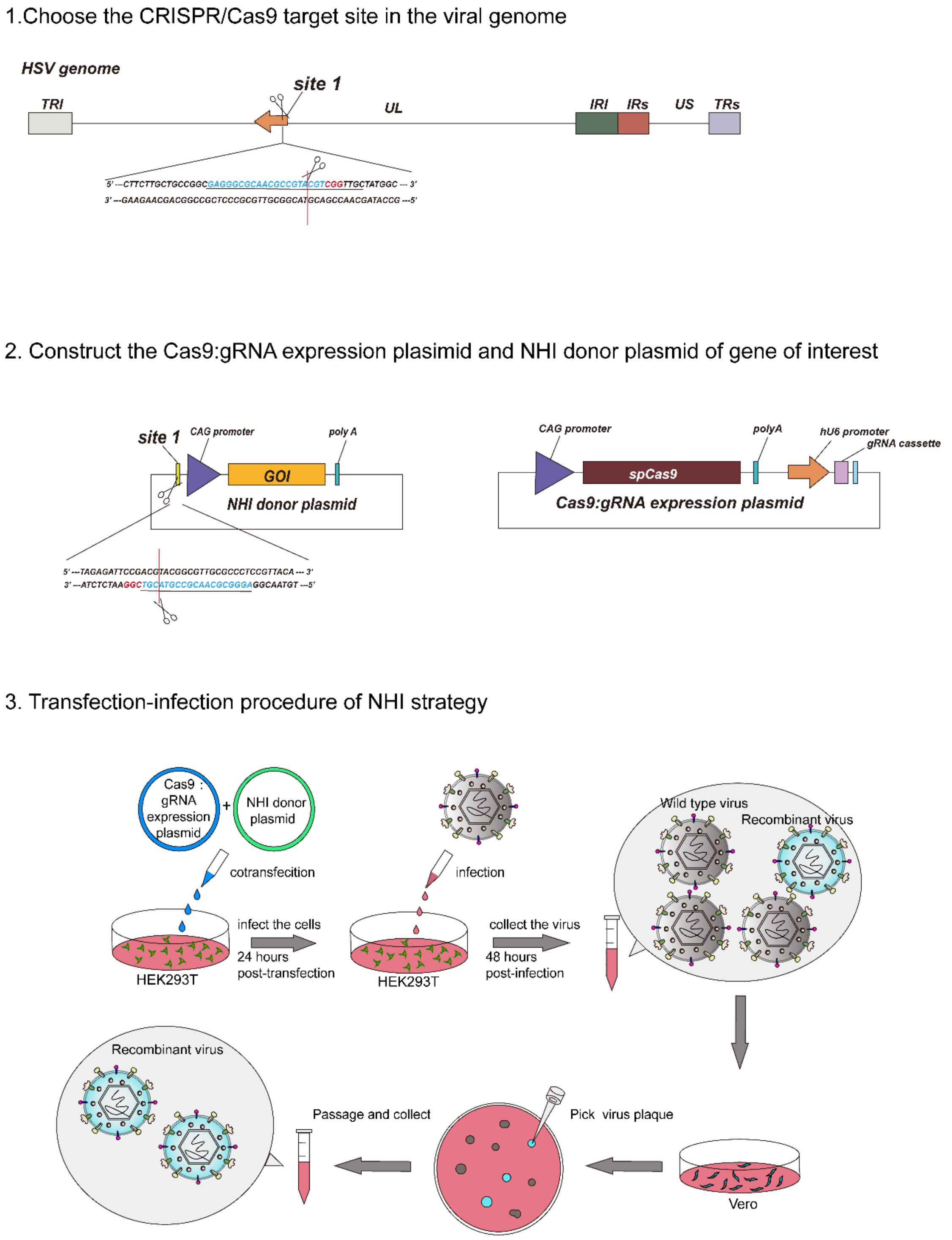

Figure S2. Main step of the NHI strategy. This schematic is previously described in figure 1 of the patent: CN108913684A.

The procedure of NHI strategy

1. choose the CRISPR/Cas9 target site in the viral genome

Using the CRISPR/Cas9 target site prediction tools (eg. CRISPOR tool, Benchling, or other available tools) to predict the potential off-target effect, editing efficiency, and rate of out-of-frame DNA repair outcomes. We select the site 1 as an example in this protocol, the base in blue indicate

the target sequence of site 1, and the base in red indicate the PAM sequence of this site, the Cas9 protein will cut the double-strand DNA at the 17th base of the target site.

2. Construct the Cas9: gRNA expression plasmid and NHI donor plasmid of the gene of interest

A. Constructing the Cas9: gRNA expression plasmid, in our experiment we select the px330 plasmid as the backbone, and other bicistronic Cas9: gRNA expression plasmid could be used.

B. Constructing the NHI donor plasmid of the gene of interest (GOI),

First, confirm the donor backbone, we select the pmd19-T vector as the backbone, the other plasmid could also be used (eg. pUC19) but considered the knock-in efficiency is associated with the length of the knock-in fragment, thus the smaller donor backbone will be efficient. Hence, the minicircle plasmid would be a better choice.

Next, confirm the GOI and the regulatory sequence, these should choose according to the purpose of the experiment. In this example, we choose the EGFP gene under the control of CMV promoter and SV40 poly A signal as our GOI.

Last and the important, the introduction of the target site into donor plasmid, in order to guarantee the donor plasmid could be recognized and cleaved simultaneously by the same Cas9: gRNA complex, thus the target site of donor plasmid should be the same as the one in the genome. However, if the target site of donor plasmid is in the same direction (eg. the target site of the genome and donor plasmid are both in the positive strand), the re-cleavage in the recombinant genome will occur due to the target site remain intact, thus in our experiment, the target site in the donor plasmid is designed as reverse complementary as the genome (middle panel in the fig S1), this will not lead to re-cleavage as the target site was broke so the GOI could insert as pre-designed direction. In addition, the target site length is 23 bp, so it could be easily introduced by PCR, then constructed into donor plasmid. It is of note that the if only one target site was introduced in the donor genome, the plasmid element will integrated into viral genome, thus, two target sites should be introduced into the donor plasmid to avoid the redundant gene insertion, or, the minicircle plasmid could be an alternative choice.

3.Transfection and infection

To begin with, the Cas9: gRNA expression plasmid and donor plasmid were co-transfected into HEK-293T cells or other permissive cells, and cells were infected at 24 hours posttransfection. Next, the cells and supernatant were harvest at 48 hours postinfection, and store at -80 °C after subjected to three freeze-thaw cycles. Then, the harvested virus was used to infect the Vero cells for 2 hours and overlaid with 1% agarose contained MEM-2 media. When the virus plaque can be observed under an inverted microscope (it typically spends ~3 days after infection), we first marked at the roof of the plate in an approximate position of the plaque, then confirm and mark the accurate plaque position at the bottom. Finally, we pick the marked plaque by stabbing the agarose layer using 10 μl pipette tips, and then the Vero cells in the 96 wells were infected with the virus by pipetting 3-5 times. When the 80% cells appear CPE, the 50 μl supernatant was used to extract the genome to perform PCR to identify the recombinant virus. when no wild-type virus could be detected, the virus in the 96 wells was harvest and used to amplify virus stock.
